# Probabilistic sunspot predictions with a gated recurrent units-based combined model guided by pinball loss

**DOI:** 10.1038/s41598-024-63878-z

**Published:** 2024-06-13

**Authors:** Zhesen Cui, Zhe Ding, Jing Xu, Shaotong Zhang, Jinran Wu, Wei Lian

**Affiliations:** 1 Department of Computer Science, Changzhi University, Changzhi, 046011 People’s Republic of China; 2 School of Computer Science, Queensland University of Technology, Brisbane, QLD 4001 Australia; 3 College of Hydraulic Science and Engineering, Yangzhou University, Yangzhou, 225009 People’s Republic of China; 4 Frontiers Science Center for Deep Ocean Multispheres and Earth System, Key Lab of Submarine Geosciences and Prospecting Techniques, MOE and College of Marine Geosciences, Ocean University of China, Qingdao, 266100 People’s Republic of China; 5 Institute for Positive Psychology and Education, Australian Catholic University, Banyo, QLD 4014 Australia

**Keywords:** Environmental sciences, Computational science

## Abstract

Sunspots play a crucial role in both weather forecasting and the monitoring of solar storms. In this work, we propose a novel combined model for sunspot prediction using improved gated recurrent units (GRU) guided by pinball loss for probabilistic forecasts. Specifically, we optimize the GRU parameters using the slime mould algorithm and employ a seasonal-trend decomposition procedure based on loess to tackle challenges related to sequence prediction, such as self-correlations and non-stationarity. To address prediction uncertainty, we replace the traditional $l_2$-norm loss with pinball loss. This modification extends the conventional GRU-based point forecasting to a probabilistic framework expressed as quantiles. We apply our proposed model to analyze a well-established historical sunspot dataset for both single- and multi-step ahead forecasting. The results demonstrate the effectiveness of our combined model in predicting sunspot values, surpassing the performance of other existing methods.

## Introduction

Predicting the amplitude of the solar cycle, along with its maximum and minimum times, is a fundamental endeavor in solar physics. Governed by the sun’s magnetic field, the solar cycle wields considerable influence over space weather dynamics, profoundly impacting Earth’s technological infrastructure^1,2^. Predicting sunspot numbers serves as a pivotal adjunct to these endeavors. Precise forecasts of sunspot numbers furnish invaluable insights into both the scale and temporal cadence of the solar cycle, facilitating prognostications regarding its zenith and nadir. Serving as a quantitative proxy for solar magnetic activity, sunspot numbers exhibit a close correlation with the amplitude and timing of the cycle^3^. Their accurate prediction not only enriches our comprehension of solar cycle dynamics but also furnishes an indispensable tool for forecasting space weather phenomena and mitigating associated hazards.

The importance of sunspot number prognostication is underscored by its correlation with various solar activity indicators, including solar flares and coronal mass ejections, which possess the capacity to disrupt satellite operations, communications, and power grids. Moreover, sunspot numbers play an integral role in elucidating the evolution of the sun’s magnetic field and the heliosphere’s response to such perturbations^4^.

From an astronomical vantage point, the precision in predicting sunspot numbers facilitates the tracing of the intricate evolution of the solar magnetic field across the solar cycle. This, in turn, aids in deciphering the underlying mechanisms governing solar activity and its manifestation in the form of sunspots^5^. Additionally, the study of sunspot numbers contributes to our comprehension of the sun’s long-term behavior and its potential impact on Earth’s climate.

The practical ramifications of sunspot number predictions are manifold, encompassing the operation of satellite-based technologies, the strategic planning of space missions, and the formulation of strategies to shield Earth’s infrastructure from the deleterious effects of solar activity. Furthermore, these prognostications hold critical relevance for the energy sector, facilitating the anticipation of heightened solar output periods that may impinge upon the efficacy of solar panels and other renewable energy technologies^6^.

The prediction of sunspot numbers constitutes an indispensable facet of solar cycle inquiry. Historically, sunspot numbers have demonstrated temporal fluctuations, characterized by cyclic patterns of heightened and diminished activity. However, recent investigations have uncovered inconsistencies in this variability, revealing long-term trends and irregularities that present challenges for accurate prediction^7–10^. The complex nature of the solar dynamo, responsible for generating magnetic fields giving rise to sunspots, adds to the uncertainties surrounding the prognosis of future sunspot activity. Despite these formidable challenges, understanding the fluctuations in sunspot numbers holds significant implications for forecasting and mitigating the impacts of solar activity on Earth’s climate and technology. Notably, increased sunspot activity can disrupt satellite communications and power grids, while reduced activity can affect Earth’s climate by reducing incoming solar energy. Therefore, predicting solar activities carries profound significance in guiding the development of various industries^11,12^.

### Literature review

Within the realm of sunspot prediction, diverse methodologies have been employed to address forecasting challenges, broadly categorized into linear and nonlinear modeling approaches^13–15^. However, the time series data representing sunspot numbers exhibits distinctive features, such as uncertainty, volatility, and cyclicity^16,17^. Hence, nonlinear modeling techniques prove more suitable for sunspot number forecasting.

Nonlinear modeling studies have extensively utilized classical statistical methods and neural network techniques for sunspot number prediction^18–20^. Moreover, combined models integrating various technologies have been employed to tackle time series forecasting challenges^21,22^.

Aggarwal et al. analyzed different time series forecasting models, including the autoregressive integrated moving average (ARIMA) model and the dynamic neural network (DNN) model, revealing that the DNN model exhibits superior time series forecasting accuracy^23^. One proposed combined model integrates ARIMA with a support vector machine (SVM) to forecast monthly and yearly sunspot numbers^24^. Zainuddin et al. introduced a modified artificial neural network (ANN)-ARIMA model, employing bootstrap methods to enhance the precision and efficiency of sunspot time series forecasting^25^. Hajirahimi et al. utilized the multi-layer perceptron model (MLP) to address the nonlinear component of the sunspot time series^26^. Pala et al. presented a combined approach that integrates the long short-term memory (LSTM) architecture and neural network autoregression for processing sunspot number time series data^27^. Moustafa et al. proposed an LSTM-ARIMA hybrid model which shows the potential of hybrid methods in improving the overall performance^28^.

### Our motivation

Building on the preceding discourse, forecasting the sunspot time series poses two prominent challenges that necessitate careful consideration and examination.

First and foremost, statistical methods encounter a predicament where stringent assumptions limit the precision of these models. Consequently, our focus is on optimizing neural network techniques, albeit with susceptibility to certain drawbacks when applied to sunspot time series analysis. These drawbacks include the risk of converging to a local minimum and the necessity for a mechanism for self-adaptive adjustment of parameters^29,30^. Hence, the significance of a hyper-parameter optimization algorithm arises to effectively determine suitable hyper-parameters for neural networks. Moreover, the sunspot time series displays distinct attributes characterized by uncertainty, volatility, and cyclicity, demanding an efficient methodology to address these issues and enhance the predictive performance of neural networks.

Secondly, the traditional approach to forecasting sunspot activity relies on point predictions, offering estimates of expected values for future solar activity^31^. However, this method falls short of capturing the inherent uncertainties associated with the complex nature of sunspot activity^32^. Hence, employing probabilistic forecasting methods becomes essential when providing comprehensive and accurate solar predictions. Despite the importance of such methods, the current literature on probabilistic forecasting for sunspot activity remains limited due to the significant uncertainties in sunspot time series data. Consequently, the predictability of sunspot activity is considerably diminished. In response, our objective is to expand upon the existing literature on quantile probabilistic forecasting and apply it to sunspot prediction.

### Our inspiration

The utilization of neural networks in solar cycle prediction and sunspot number forecasting carries considerable significance^33^. Notably, the employment of the GRU model for sunspot number prediction has yielded valuable insights^34^. The employment of meta-heuristic optimization algorithms has demonstrated promise in efficiently optimizing neural network parameters^35,36^. These algorithms provide an effective and flexible means of exploring high-dimensional search spaces while avoiding local optima, thereby preventing suboptimal solutions^37^. The integration of meta-heuristic optimization techniques into neural network parameter tuning has shown significant enhancements in overall performance and accuracy^38^. Therefore, employing meta-heuristic optimization algorithms stands as a valuable strategy for improving the efficiency of neural networks.

The time series decomposition method has emerged as an effective approach for addressing challenges posed by uncertainty, volatility, and periodicity in time series data^39,40^. By decomposing a time series into its various components, such as trend, seasonality, and residual, this method facilitates a comprehensive understanding of underlying patterns and fluctuations within the data. The ability to separate and analyze different components enables more accurate predictions and informed decisions in the realm of time series forecasting.

Quantile probabilistic forecasting involves generating predictions that explicitly account for uncertainty by producing a full probability distribution for future outcomes^41,42^. Instead of providing a single-point estimate, probabilistic forecasting offers a range of possible values along with associated probabilities, reflecting the inherent uncertainty in the prediction process.

### Our contribution

Following our motivations, with the inspiration mentioned before, we present a novel combined model, GRU-SMA-STL guided by pinball loss, for probabilistic forecasts for sunspots. In detail, the primary $\textbf{contributions}$ of this study are outlined as follows:

- Introduction of a combined model, GRU-SMA-STL, designed for forecasting the progression of sunspot numbers. Specifically, GRU, a neural network model suitable for time series forecasting, is employed; the SMA optimizer determines optimal weight parameters, encompassing batch size and the number of neurons in GRU; and STL is utilized to extract crucial features.
- Proposal of an innovative probabilistic forecasting approach for sunspot time series by integrating GRU-SMA-STL with a pinball loss function. This model incorporates a quantile parameter to generate a comprehensive probability distribution for predictions.
- Verification of the effectiveness and superiority of the proposed quantile model through evaluations for both single-step ahead and multi-step ahead predictions on the sunspot dataset. Specifically, both SMA and STL techniques contribute to enhancing the forecasting performance of the GRU model. Additionally, the GRU-SMA-STL model guided by pinball loss generates probabilistic sunspot forecasts with lower uncertainty levels than the baseline model.

## Prerequisites

### GRU

**Figure 1:**
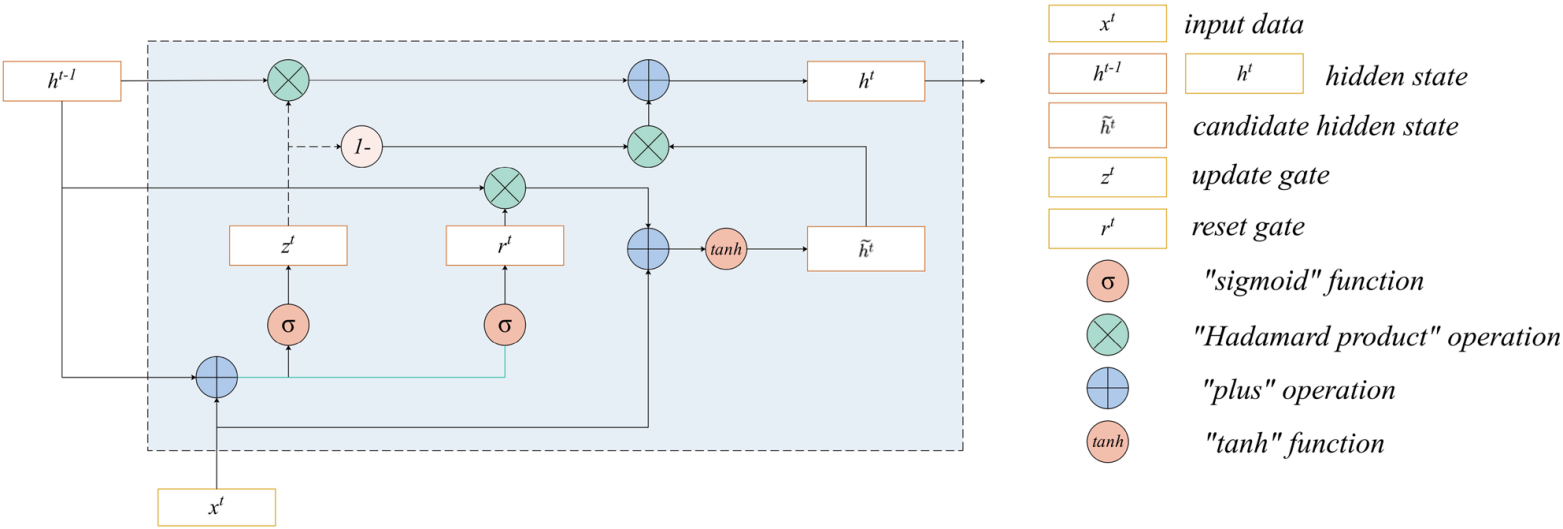
The internal architecture of the GRU model.

GRU^43^ is suggested as a solution to issues related to long-term memory and backpropagation gradients, offering higher training efficiency than LSTM. The primary architecture is illustrated in Fig. 1. The internal architecture of the GRU comprises various components. The GRU incorporates gating mechanisms, including the reset and update gates, which regulate the flow of information. These gates enable the GRU to selectively retain and update relevant information while discarding unnecessary data, thereby mitigating the vanishing gradient problem. Additionally, the GRU has internal states that encode and maintain information over sequential inputs, allowing for the capture of long-term dependencies in the data.

#### Update gate and reset gate

GRU incorporates distinct reset and update gates, expressed by:

1 $$\begin{aligned} z_{j} = \sigma ([xW_{z}]_j+[h^{t-1}U_{z}]_j), \end{aligned}$$

and

2 $$\begin{aligned} r_{j} = \sigma ([xW_{r}]_j+[h^{t-1}U_{r}]_j). \end{aligned}$$

The update gate $z_{j}^{t}$ determines the extent to which information from the previous hidden layer state is passed to the current hidden state $h^{t}$. When the parameter $z_{j}$ approaches zero, values related to the previous hidden layer state are disregarded in the current hidden layer, only being retained when $z_{j}$ approaches one.

Conversely, the reset gate $r_{j}^{t}$ dictates the amount of information from the previously hidden layer that should be forgotten. A value of $r_{j}$ near zero implies that information from the previous moment is discarded in the current memory content.

While the update gate influences the hidden state at the previous moment and, consequently, the hidden state of the current moment, the reset gate operates on the current memory content.

#### Determine the current memory content

The present candidate $\widetilde{h_{j}^{t}}$ represents crucial information recorded by GRU, comprised of two components. One part encapsulates vital information about the past, stored within the reset gate. The other part encompasses information from the current moment. The formula is presented as:

3 $$\begin{aligned} \widetilde{h_{j}^{t}} = \text {tanh}([x^tW]_j+[(r^t\odot h^{t-1})U]_j). \end{aligned}$$

#### Determine retained information in the hidden layer

The final value $h_{j}^{t}$ is generated according to:

4 $$\begin{aligned} h_{j}^{t}=z_{j} h_j^{t-1}+(1-z_{j}) \widetilde{h_{j}^{t}}. \end{aligned}$$

### SMA

SMA^44^ is a novel metaheuristic optimization technique inspired by the foraging behavior of slime moulds. This algorithm is designed to simulate the process of slime mould foraging, which involves the creation of a network of veins that connect food sources in an efficient manner. The SMA leverages this natural process to solve optimization problems by adapting the behaviors of slime mould to the search for optimal solutions in a given problem space. Slime moulds are unique organisms that exhibit both amoeboid and filamentous structures. They are known for their ability to navigate complex environments in search of food, creating efficient networks that connect multiple food sources. The parameters in the SMA and their explanations are shown in Table 1.

**Table 1:** Parameters explanation for SMA.

Parameters	Description
*pop*	The number of slime mould entities in the search space, each representing a potential solution to the optimization problem
$Max\_iter$	The maximum number of iterations the algorithm will perform
$X_i$	The position of a slime mould entity in the *n*-dimensional search space, where *n* corresponds to the number of variables in the optimization problem
*t*	The current iteration within the algorithm. Each iteration corresponds to a cycle during which the slime mould agents evaluate their positions and update them
*W*	The weight associated with each slime mould entity, influences its movement and decision-making within the search space
$X_b$	The position of the slime mould entity with the highest odor concentration, representing the most promising solution found so far
*vb*	A value that varies within a specified range, is used to introduce randomness and exploration into the search process
*vc*	A value that linearly decreases from one to zero, is used to adjust the exploration-exploitation balance during the iterations
*S*(*i*)	The fitted value of $X_i$, which is the quality or fitness of the solution represented by the position $X_i$
*DF*	The best solution found at each iteration
*LB*, *UB*	The lower and upper boundaries of the search range, define the limits of the search space
*rand*, *r*	Random values within a specified range, are used to introduce stochastic elements to the algorithm
$X_{A}, X_{B}$	Two distinct slime mould entities, are used in the algorithm for comparison and decision-making purposes

The SMA optimizes the number of neurons and batch size for the GRU by framing the hyperparameter selection as a search problem. Each slime mould agent represents a set of hyperparameters, including the neuron count and batch size. The agents’ fitness is evaluated based on the GRU’s performance metric, such as validation accuracy. SMA iteratively refines the agent positions, simulating slime mould foraging behavior to balance exploration and exploitation in the hyperparameter space. The agent with the highest fitness after a series of iterations indicates the optimal configuration for the GRU’s neurons and batch size, aiming to maximize the model’s predictive performance. The SMA optimizer works by iteratively updating the positions of the slime mould entities based on their current fitness and the feedback from the search space. The algorithm balances exploration (searching new areas) and exploitation (refining existing solutions) by adjusting the weights and positions of the entities. Over time, this process converges toward an optimal solution.

The algorithm is divided into three main steps: approach food, wrap food, and grabble food, which mimic the mould’s process of seeking, engulfing, and consuming food sources.

#### Approach food

This step models the slime mould’s attraction toward food sources based on the concentration of nutrients. The mould approaches the food by moving towards locations with higher odor concentration, which corresponds to better fitness values in the optimization context. The movement is governed by the formula:

5 $$\begin{aligned} X(t+1)=\left\{ \begin{matrix} {X_{b}(t)}+{vb} \cdot ( {W} \cdot {X_{A}(t)} - {X_{B}(t)} ), r<p,\\ {vc} \cdot {X(t)}, r\ge p. \end{matrix}\right. \end{aligned}$$

The formula for *p* is given by,

6 $$\begin{aligned} p = \tanh \left| S(i) - DF \right| , \end{aligned}$$

where $$i\in 1,2,\ldots ,n$$.

#### Wrap food

Once the slime mould reaches a food source, it wraps the food by adjusting its search patterns based on the quality of the food found. The computation of each slime mould’s location is defined as,

7 $$\begin{aligned} {X^{*}}=\left\{ \begin{matrix} rand\cdot (UB-LB)+LB, rand<z,\\ {X_{b}(t)}+{vb} \cdot ( {W} \cdot {X_{A}(t)} - {X_{B}(t)} ), r<p,\\ {vc} \cdot {X(t)}, r\ge p. \end{matrix}\right. \end{aligned}$$

#### Grabble food

The final step, grabbing food, simulates the mould’s decision to either stay at the current food source or search for better ones. This is achieved by comparing the fitness values of the current position with a random value. If the current position is better, the mould grabbles the food and stays put; otherwise, it moves to a new location.

The SMA is outlined in Algorithm 1.

**Algorithm 1:**
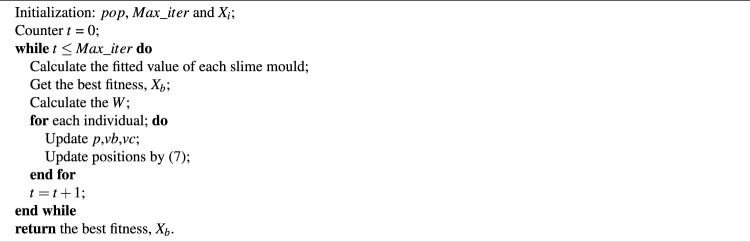
**SMA.**

### STL

STL^45^ is a time series decomposition algorithm grounded in loess, aimed at breaking down the variable $Y_{v}$ into its constituent components: the trend component $T_{v}$, the seasonal component $S_{v}$, and the remainder component $R_{v}$. This decomposition is expressed by:

8 $$\begin{aligned} Y_{v}=T_{v}+S_{v}+R_{v}, v=1,\ldots ,N. \end{aligned}$$

STL comprises an inner loop and an outer loop. In the inner loop, the primary focus is on calculating trend fitting and periodic components. Assuming $T_{v}^{(k)}$ and $S_{v}^{(k)}$ represent the trend component and periodic component, respectively, after the $(k-1)$th iteration within the inner loop.

The inner loop of STL is outlined in Algorithm 2. The outer loop is predominantly responsible for regulating the robustness of weight. The parameter *h* is defined as 6 times the median of $|R_{v}|$. The formula for the robustness weight is specified as $\rho {v}=B(|R{v}|/h)$, with the bisquare function *B*(*u*) defined as:

9 $$\begin{aligned} B(u)= \left\{ \begin{matrix} (1-u^2)^2 \quad for \quad 0 \le u <1,\\ 0 \quad for \quad u \ge 1 . \end{matrix}\right. \end{aligned}$$

**Algorithm 2:**
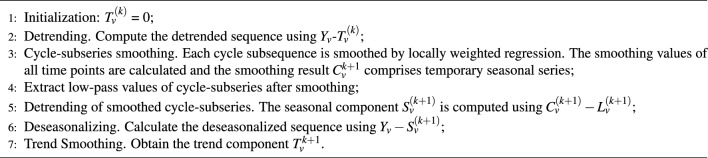
**The inner loop for STL.**

### Pinball loss

The $l_2$-norm loss exhibits sensitivity to outliers. In many practical prediction scenarios, the desire is often to capture the uncertainty in predictions. Predicting an interval of values, as opposed to a singular point, becomes crucial for informed decision-making in specific business processes.

The pinball loss function emerges as a valuable tool when there is a need to predict the value interval of the outcome, demonstrating robust performance even with non-uniformly distributed residuals. In the case of the traditional GRU, the loss function is $l_2$-norm loss, expressed by:

10 $$\begin{aligned} L_{MSE} = \frac{1}{m}\sum _{i=1}^{m}(\widehat{y}_{i}- {y}_{i} )^{2} , \end{aligned}$$

where $\widehat{y}_{i}$ and ${y}_{i}$ denote the forecasted and observed sunspot values at time *i*, respectively, and *m* represents the total length of the forecasting time series.

**Figure 2:**
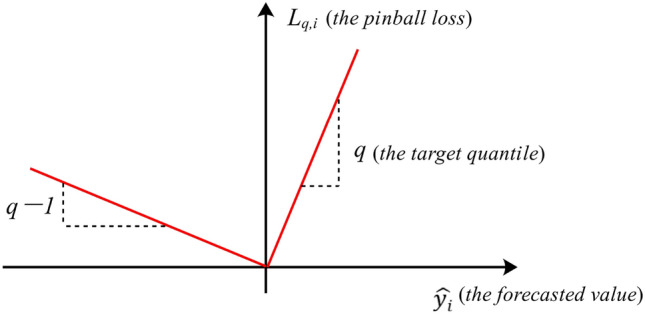
Illustration of pinball loss.

Traditional GRU is limited to predicting the expected sunspot value in the future. To convey more uncertainty in predictions, the pinball loss replaces $l_2$-norm as the new loss function, contributing to enhanced training accuracy for GRU. The pinball loss function is defined as:

11 $$\begin{aligned} L_{q,i}({y}_{i} ,\widehat{y}_{i}) ={\left\{ \begin{array}{ll} q( {y}_{i} -\widehat{y}_{i}) & \widehat{y}_{i}<{y}_{i}, \\ (1-q)(\widehat{y}_{i}- {y}_{i} ) & \widehat{y}_{i}\ge {y}_{i}, \end{array}\right. } \end{aligned}$$

where *q* represents the target quantile, ${y}_{i}$ and $\widehat{y}_{i}$ represent the observed value and quantile forecasting value at time *i*, respectively. The visual representation of this loss function is depicted in Fig. 2. This function quantifies the loss resulting from deviations between predicted quantiles and actual observed values. The horizontal axis illustrates the forecasted values, which serve as the model’s predictive outputs. This axis lists the potential outcomes predicted by the model. Each value corresponds to a possible estimate of the variable of interest. On the vertical axis lies the pinball loss, measuring the disparity between forecasted values and the target quantile *q*. This axis quantifies the loss associated with each forecasted value, with lower values indicating more precise predictions that closely match the actual observations.

The asymmetric design of the pinball loss function reflects the varying implications of underestimation and overestimation. The function attains zero when the forecasted value precisely aligns with the target quantile, signifying a flawless prediction. As the forecasted values diverge from the target, the loss escalates. When the quantile forecasting value surpasses the observed result, the loss value is influenced by the parameter $(1-q)$; conversely, when the quantile forecasting value is lower than the observed result, the loss value is influenced by the parameter *q*. This deliberate asymmetry aligns with the cost-sensitive nature of specific forecasting contexts. The graph’s characteristic V-shape accentuates the significance of accurate quantile predictions, highlighting the heightened sensitivity of the loss function to deviations around the target value.

In practice, the pinball loss function is used to train models to predict quantiles of the target variable. By optimizing this loss function, the model learns to estimate a range of possible values at different confidence levels, beyond just the mean prediction. Overall, the pinball loss function provides a robust way to assess the performance of quantile regression models.

The merits of the pinball loss function can be delineated as follows:

- The pinball loss function guides the trained GRU model to derive the anticipated quantile forecasting value. Altering the quantile values facilitates the representation of various levels of uncertainties without the need for assumptions about the distribution throughout the entire training process.
- The pinball loss serves as a comprehensible composite index, encompassing reliability, sharpness, and calibration. This characteristic contributes to improved performance in probabilistic forecasting.

## Proposed model

### GRU-SMA-STL

This section introduces the established model GRU-SMA-STL. To elaborate, the STL is applied to decompose the original data, and the SMA is employed to obtain suitable parameters for the GRU. Subsequently, GRU is trained and tested on the trend component, seasonal component, and remainder component of the sunspot dataset. The proposed GRU-SMA-STL model is provided in Algorithm 3.

**Algorithm 3:**
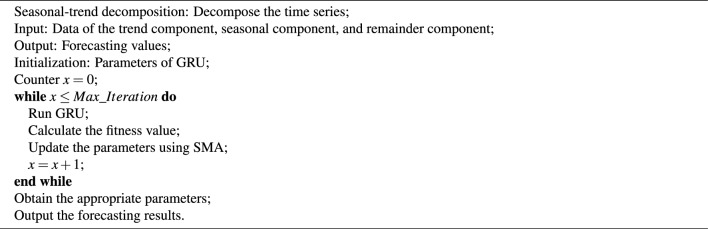
**The GRU-SMA-STL.**

The procedural steps can be outlined as follows:

Step 1: The original time series undergoes decomposition into three components using STL.

Step 2: SMA is employed to initialize key parameters, including batch size, and the number of neurons in the hidden layer.

Step 3: To establish the fitness function, the root mean squared error (RMSE) is chosen, defined by:

12 $$\begin{aligned} \text{ RMSE }=\sqrt{\frac{1}{m} \sum _{i=1}^{m} ({\hat{y}_{i}-y_{i}} )^{2}}, \end{aligned}$$

where $y_{i}$ represents the observed value, and $\hat{y}_{i}$ is the forecasting value.

Step 4: In each iteration, future values are forecasted based on the data of the trend component, seasonal component, and remainder component. Parameters are updated according to the fitness value. Once iterations are completed, Step 5 follows.

Step 5: The optimal parameters yielding the minimum fitted results in GRU, along with the forecasting outcomes, are obtained.

### GRU-SMA-STL guided by pinball loss

**Figure 3:**
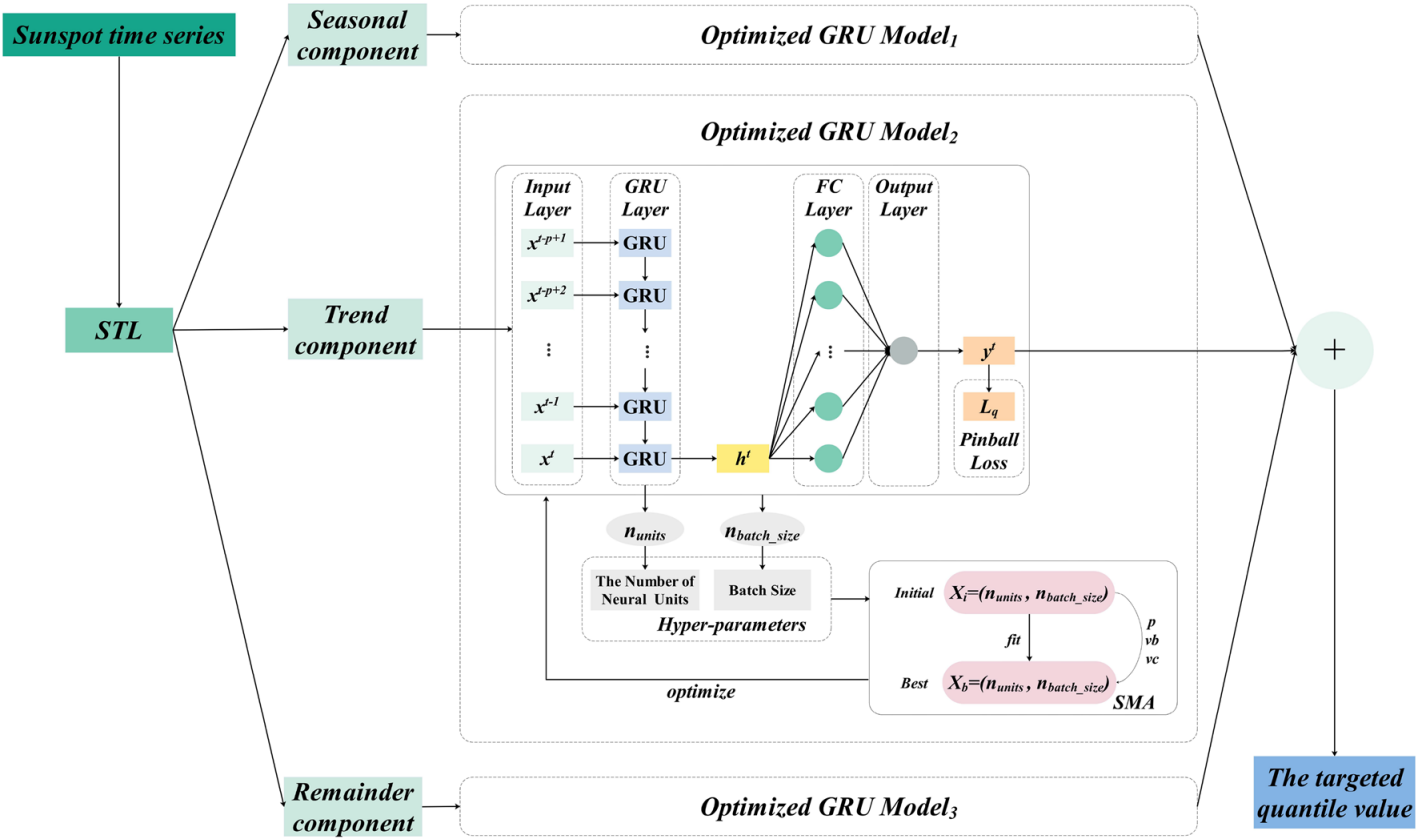
Overall architecture of pinball loss-guided GRU-SMA-STL.

**Figure 4:**
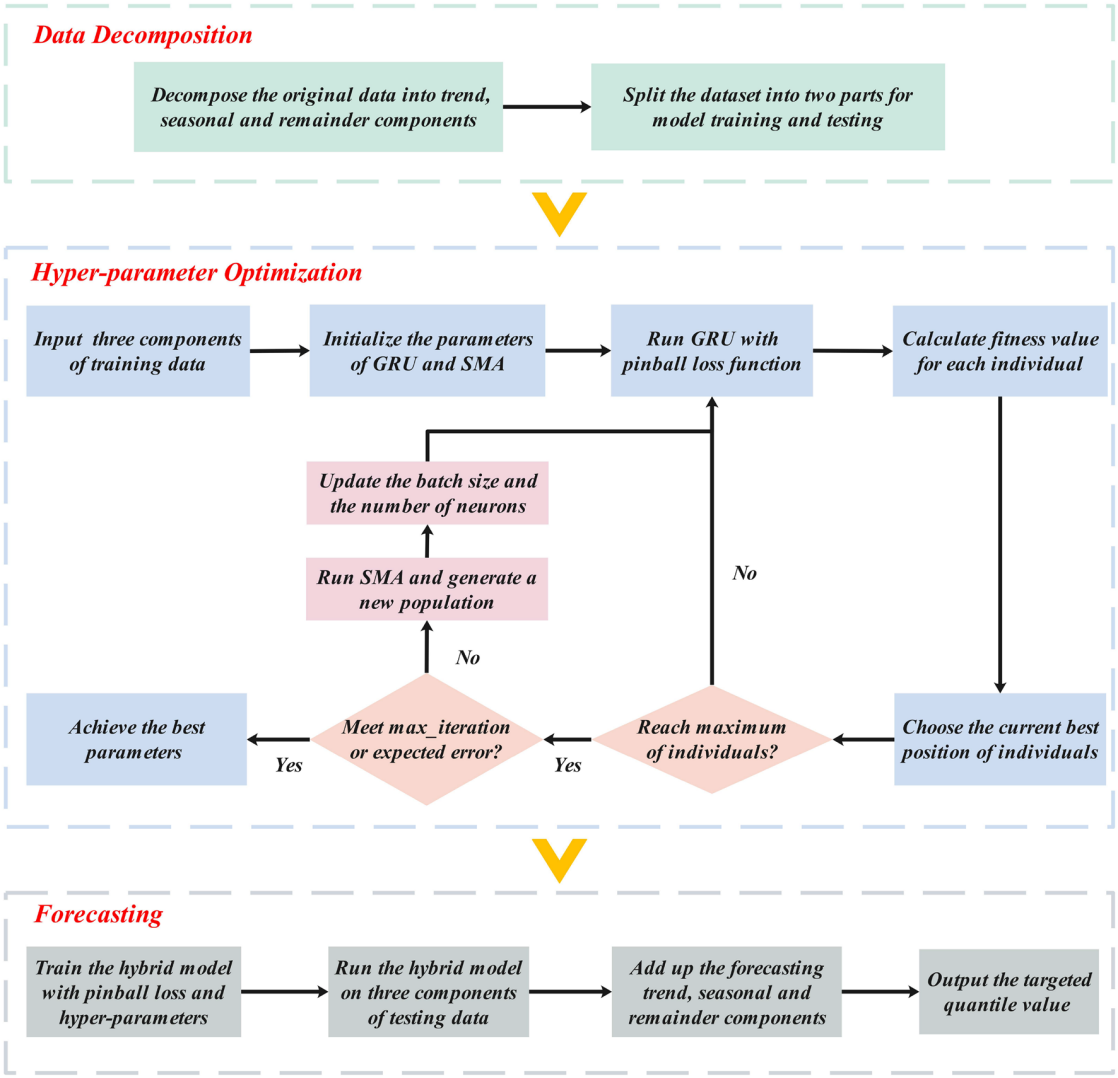
Flowchart for implementing probabilistic sunspot time series forecasting.

This study introduces an innovative framework for probabilistic sunspot number forecasting in the form of quantiles, denoted as the pinball loss-guided GRU-SMA-STL, illustrated in Fig. 3. The framework combines the strengths of GRU-SMA-STL and the pinball loss. Specifically, GRU-SMA-STL captures both long- and short-term dependencies within sunspot data, while the pinball loss imparts valuable future uncertainty information through predefined quantiles. By integrating these two techniques, the proposed method can deliver precise probabilistic forecasts for sunspot numbers. The framework initially decomposes the input sunspot time series into three distinct datasets: the trend component, the seasonal component, and the remainder component. Subsequently, each component undergoes processing by a corresponding GRU model optimized by SMA. These models utilize the pinball loss function. The predictions produced by these models are then aggregated to formulate the final forecast for quartile *q*. Through the extraction of prediction outcomes corresponding to various quartiles, a range of diverse prediction intervals can be obtained. The flowchart illustrating sunspot forecasting using GRU-SMA-STL guided by the pinball loss function is presented in Fig. 4. In this section, a more detailed predictive process is outlined. The implementation of probabilistic sunspot forecasting employs a three-stage approach. Initially, the data decomposition phase involves decomposing the sunspot dataset into three components. Subsequently, the dataset is divided into training and testing subsets for model development. In the hyper-parameter optimization phase, the suitable parameters for the pinball loss-guided GRU are determined through SMA. In the third phase, the optimized model is trained and tested to generate the target quantile forecasting results. Overall, this approach holds promise for enhancing the accuracy and reliability of sunspot forecasting.

## The case study

### The data collection and analysis

**Figure 5:**
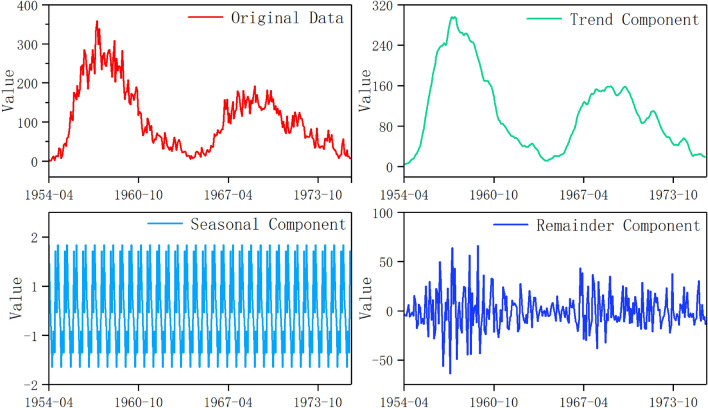
The monthly sunspot time series and its three decomposed subsequences.

Concerning the sunspot dataset, the variability, and irregularity inherent in the sunspot time series pose challenges to the forecasting process. To enhance forecasting accuracy, the sunspot time series undergoes decomposition into three components using STL. The segmented original sunspot data and the resulting decomposed subsequences are illustrated in Fig. 5. More precisely, the trend component elucidates the prolonged trends within the sunspot time series, indicating overarching increases or decreases over time. It captures sustained changes and fundamental patterns that transcend short-term fluctuations. Conversely, the seasonal component delineates regular, cyclic fluctuations transpiring within fixed time intervals. This component plays a pivotal role in discerning and accommodating seasonal behaviors, thereby facilitating a more precise data analysis during specific time frames. Meanwhile, the remainder component encompasses random variations unaccounted for by the trend or seasonal patterns, underscoring the influence of unpredictable factors and inherent data noise. These three components serve as the foundation of the original sunspot time series, with the cumulative sum of the trend, seasonal, and remainder components adeptly reconstructing the series with remarkable accuracy.

The novel proposed model, GRU-SMA-STL, is assessed utilizing the monthly sunspot time series dataset spanning from February 1755 to December 2019, covering all 24 solar cycles. This evaluation encompasses both single-step ahead and multi-step ahead predictions. The experimental datasets are partitioned into two distinct segments: training and test. The initial 18 solar cycles, from February 1755 to March 1954, are utilized for model training, while the subsequent 6 cycles, from April 1954 to December 2019, are reserved for testing purposes, enabling the rigorous assessment of the model’s predictive efficacy. All datasets are obtained from the SILSO website (www.sidc.be/silso/datafiles).

### Experimental design and evaluation criterion

#### The process of forecasting

The predictions for both single-step ahead and multi-step ahead scenarios are detailed as follows: when employing a sliding window size of *p*, the last *p* sunspot numbers serve as input for GRU-SMA-STL. For one-step ahead forecasting, the final result is denoted as $x_{n}$. The corresponding mapping relationship is expressed as:

13 $$\begin{aligned} (x_{n-p},x_{n-p+1},\ldots ,x_{n-2},x_{n-1})\rightarrow (x_{n}). \end{aligned}$$

For two-step ahead forecasting, the final result is represented by $x_{n},x_{n+1}$. The calculation formula is articulated as:

14 $$\begin{aligned} (x_{n-p},x_{n-p+1},\ldots ,x_{n-2},x_{n-1})\rightarrow (x_{n},x_{n+1}). \end{aligned}$$

Concerning three-step ahead forecasting, the final result is denoted as $x_{n},x_{n+1},x_{n+2}$. The corresponding calculation formula is expressed as:

15 $$\begin{aligned} (x_{n-p},x_{n-p+1},\ldots ,x_{n-2},x_{n-1})\rightarrow (x_{n},x_{n+1},x_{n+2}). \end{aligned}$$

#### Evaluating GRU-SMA-STL

The performance of the GRU-SMA-STL model is compared against several baseline models, including ARIMA, recurrent neural network (RNN)^46^, MLP^47^, GRU, RNN-STL, MLP-STL, GRU-STL, Elman Artificial Neural Network (ElmanANN)^48^, and WaveNet-Long Short Term Memory (WaveNet-LSTM)^33^. ARIMA, a generalized autoregressive moving average model, predicts future points in the series. Its main parameter settings include a lag order of 4 and a moving average order of 4. RNN is a type of neural network that processes various time series using internal state memory. MLP, belonging to feedforward neural networks, comprises an input layer, a hidden layer, and an output layer. MLP utilizes backpropagation for training datasets and can distinguish nonlinearly separable data. The ElmanANN represents a form of recurrent neural network specifically tailored to address temporal data, owing to its intrinsic feedback connections originating from the hidden layer to itself. This mechanism facilitates the retention of past computations, endowing the network with a memory element. The configuration of the Elman ANN employed in this investigation encompasses an input layer, a hidden layer, and an output layer. The WaveNet-LSTM constitutes a hybrid deep learning framework merging the autoregressive essence of WaveNet with the long-term memory functionalities inherent in LSTM networks, devised primarily for time series forecasting. Notable parameters of the model encompass the count of convolutional kernels set at 4, a kernel size of 2, alongside dilated convolutional layers featuring exponentially escalating dilation rates. For RNN, MLP, GRU, RNN-STL, MLP-STL, GRU-STL, ElmanANN, and WaveNet-LSTM, the sliding window size is set to 12, the number of neurons in the hidden layer is set to 100, the batch size is set to 64, the epoch is set to 100, and the learning rate is set to 0.0001. For GRU-SMA-STL, both the number of neurons and batch size are determined using the SMA optimizer. The parameters mentioned above are summarized based on many experiments, resulting in finely tuned parameters.

Meanwhile, the evaluation criterion, including mean absolute error (MAE), RMSE, symmetric mean absolute percentage error (SMAPE), R-squared ($R^{2}$), and Adjusted R-squared ($R_{adj}^{2}$) are employed to evaluate the point forecasting experimental results. The formulas are presented as,

16 $$\begin{aligned} \text{ MAE }&= \frac{1}{m} \sum _{i=1}^{m} |{\hat{y}_{i}-y_{i}}|, \end{aligned}$$

17 $$\begin{aligned} \text{ RMSE }&= \sqrt{\frac{1}{m} \sum _{i=1}^{m}({\hat{y}_{i}-y_{i}} )^{2}}, \end{aligned}$$

18 $$\begin{aligned} \text{ SMAPE }&= \frac{100\%}{m} \sum _{i=1}^{m}\frac{|{\hat{y}_{i}-y_{i}}| }{(|\hat{y}_{i}|+|y_{i}|)/2}, \end{aligned}$$

19 $$\begin{aligned} R^{2}&= 1-\frac{\sum _{i=1}^m (\hat{y}_i - y_i )^2}{\sum _{i=1}^m (\bar{y}_i - y_i )^2}, \end{aligned}$$

and

20 $$\begin{aligned} R_{adj}^{2} = 1-\frac{(1-R^{2})(m-1)}{(m-p-1)} , \end{aligned}$$

where *m* is for the sample size, $\hat{y}_i$ for the prediction, $\bar{y}_i$ for the average value, $y_i$ for the observation, and *p* for the number of predictor variables.

#### Evaluating GRU-SMA-STL guided by pinball loss

The performance of the GRU-SMA-STL model using pinball loss is compared against the baseline quantile regression method. The hyper-parameter setting involves the use of a sliding window size of 12. The capability of the probabilistic forecasts is evaluated by the average of the total pinball loss:

21 $$\begin{aligned} L_{avg} = \frac{1}{Q*S} \sum _{q\in Q} \sum _{i\in S}L_{q,i}({y}_{i} ,\widehat{y}_{i}^{q}), \end{aligned}$$

where *Q* denotes all the quantiles, *S* denotes the length of the test dataset, *q* denotes the targeted quantile, $\widehat{y}_{i}^{q}$ denotes the estimated *q*th quantile forecasting value at time *i*, and $L_{q,i}$ denotes the pinball loss for the *q*th quantile at time *i*.

### Discussion of results

#### Discussion of GRU-SMA-STL

Each experiment was repeated 10 times using the sunspot dataset. Table 2 presents the average evaluation results for single-step ahead and multi-step ahead predictions from various models, including ARIMA, RNN, MLP, GRU, RNN-STL, MLP-STL, GRU-STL, ElmanANN, WaveNet-LSTM, and our proposed GRU-SMA-STL model. Notably, the GRU-SMA-STL model exhibited superior performance among the different models. Specifically, our proposed model achieved the best results in terms of MAE, RMSE, SMAPE, $R^{2}$, and $R_{adj}^{2}$.

As depicted in Table 2, the robustness of the compared models decreases as the prediction horizon increases. The evaluation results for MAE, RMSE, and SMAPE indicate that single-step-ahead forecasting yields lower values compared to multi-step-ahead forecasting. Furthermore, $R^{2}$ and $R_{adj}^{2}$ for single-step ahead forecasting are higher than those for multi-step ahead forecasting, suggesting greater accuracy in the former. This discrepancy can be attributed to the growing uncertainties associated with multi-step prediction. However, our GRU-SMA-STL model consistently performs well in both single-step ahead and multi-step ahead forecasting, as evidenced by the similar evaluation results for MAE, RMSE, SMAPE, $R^{2}$, and $R_{adj}^{2}$. This suggests that our proposed model effectively manages the increasing uncertainties as the prediction horizon expands.

**Table 2:** Evaluation indexes of different models for single-step ahead and multi-step ahead sunspot number forecasting.

	Method	MAE	RMSE	SMAPE	$R^{2}$	$R_{adj}^{2}$
one-step	ARIMA	19.1936	25.2903	36.6455	0.8900	0.8899
	RNN	17.5591	24.3279	31.6000	0.8981	0.8980
	MLP	19.0093	26.2581	33.8345	0.8812	0.8810
	GRU	17.3609	24.1638	31.8805	0.8995	0.8993
	RNN-STL	15.4159	20.6570	33.8986	0.9249	0.9248
	MLP-STL	17.1654	23.8357	30.8258	0.9022	0.9021
	GRU-STL	15.3679	20.1509	29.7838	0.9299	0.9298
	ElmanANN	17.8010	24.4142	32.4293	0.8974	0.8972
	WaveNet-LSTM	17.4356	24.2663	31.6357	0.8986	0.8985
	GRU-SMA-STL	13.7286	19.0557	25.7129	0.9374	0.9373
two-step	ARIMA	21.5401	28.0915	39.6377	0.8643	0.8641
	RNN	18.9841	26.2977	33.2245	0.8809	0.8807
	MLP	20.3627	28.1220	72.0478	0.8636	0.8634
	GRU	18.7862	26.1892	33.7671	0.8819	0.8817
	RNN-STL	15.5571	20.7216	32.0892	0.9258	0.9257
	MLP-STL	18.4907	25.5715	65.1656	0.8874	0.8873
	GRU-STL	15.6474	20.5750	29.8559	0.9269	0.9268
	ElmanANN	18.9558	26.0349	33.5795	0.8833	0.8831
	WaveNet-LSTM	18.7953	25.9606	62.9484	0.8834	0.8833
	GRU-SMA-STL	13.8988	19.3829	26.3074	0.9352	0.9351
three-step	ARIMA	23.4616	30.2863	41.8371	0.8422	0.8420
	RNN	19.9475	27.3835	34.3581	0.8708	0.8706
	MLP	20.3165	28.0509	107.9392	0.8642	0.8640
	GRU	19.7404	27.2696	33.7557	0.8719	0.8717
	RNN-STL	15.7562	20.7204	31.1071	0.9257	0.9256
	MLP-STL	19.3955	26.8013	100.4035	0.8762	0.8761
	GRU-STL	15.7172	20.5318	29.9441	0.9270	0.9269
	ElmanANN	19.8186	27.3683	34.3489	0.8709	0.8708
	WaveNet-LSTM	19.6195	27.0355	73.2739	0.8734	0.8732
	GRU-SMA-STL	13.9520	19.3117	25.9174	0.9356	0.9355

**Figure 6:**
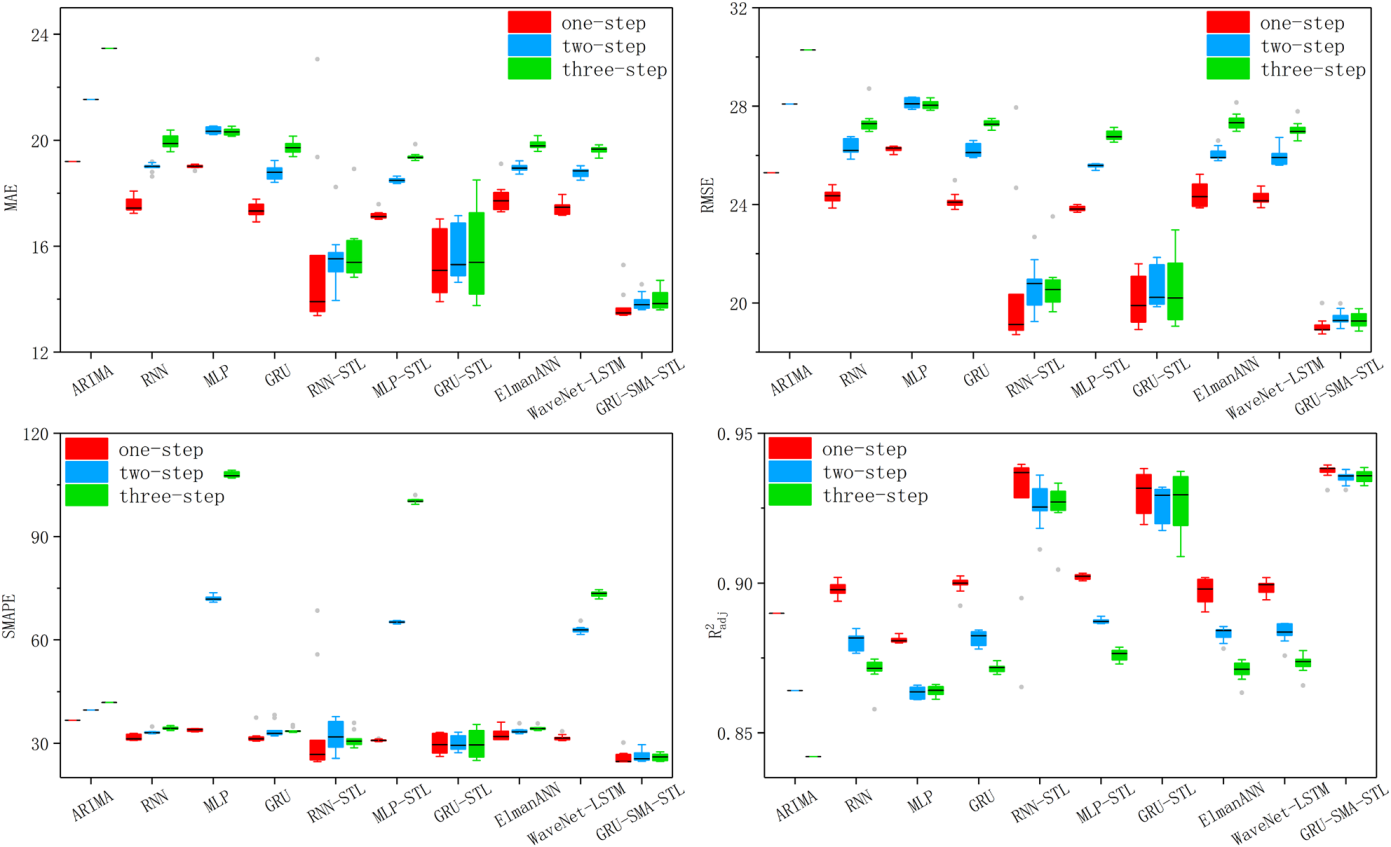
Boxplots of experimental results for one-step ahead, two-step ahead, and three-step ahead forecasting.

Upon delving into the outcomes of various models in the stationary predictive step, our analysis reveals that neural network models, including RNN and GRU, consistently outperform the statistical ARIMA model, irrespective of whether it involves single-step ahead or multi-step ahead forecasting. MLP, on the other hand, demonstrates superiority in terms of MAE and RMSE, albeit exhibiting a lower SMAPE value specifically in two-step ahead and three-step ahead forecasting. These findings collectively suggest that neural network models, as opposed to the ARIMA model, prove more suitable for forecasting non-stationary sunspot time series.

By focusing on the outcomes of models integrated with STL and SMA adjustment, respectively, our investigation underscores the enhanced forecasting performance resulting from combining decompositions and global optimal methods. The study demonstrates that the neural network optimized by STL outperforms its counterpart without STL, showcasing the efficiency of STL in addressing the cyclicity inherent in sunspot time series data. When comparing GRU-STL and GRU-SMA-STL, the latter consistently exhibits superior performance, emphasizing that SMA plays a pivotal role in acquiring hyperparameters that enhance the effectiveness of sunspot time series forecasting. The evaluation criteria values for single-step ahead and multi-step ahead forecasting further validate the broad applicability of STL and SMA. STL’s capability to decompose the sunspot dataset into efficient components enables neural networks to extract crucial features effectively, while SMA facilitates the acquisition of hyperparameters that contribute to heightened forecasting accuracy. Among the optimized models, GRU-STL demonstrates superior prediction ability compared to the others. This superiority may be attributed to GRU’s ability to address gradient vanishing and explosion issues, allowing it to learn long-term dependencies. Additionally, the model’s fewer parameters reduce the risk of overfitting. When benchmarking GRU-SMA-STL against ElmanANN and WaveNet-LSTM, our experimental results demonstrate that GRU-SMA-STL outperforms these two leading approaches in terms of performance metrics. This superior performance can be attributed to the synergistic integration of GRU, SMA, and STL, collectively enhancing the model’s capability to capture complex temporal dynamics and structural patterns within the data.

In neural network experimentation, variability in forecasting results arises from inherent randomness, which leads to changes in performance metrics. To address this and gain a comprehensive understanding of model performance, we employed box plots to visualize the distribution of key metrics, such as MAE, RMSE, SMAPE, and $R_{adj}^{2}$ for various prediction steps across ten experiments. Figure 6 depicts box plots illustrating the median, quartiles, and data spread for each performance metric across various models. These plots enable the assessment of central tendency and variability.

Comparing models, GRU-SMA-STL exhibits lower median MAE, RMSE, and SMAPE values than the nine compared models, indicating its superior predictive accuracy. Its narrower interquartile range (IQR) suggests less variability and greater consistency compared to RNN-STL and GRU-STL. Additionally, GRU-STL displays higher variability, possibly due to sensitivity to initial conditions. Conversely, GRU-SMA-STL exhibits lower variability, suggesting that SMA enhances prediction stability for GRU-STL. Furthermore, GRU-SMA-STL demonstrates a higher median $R_{adj}^{2}$, indicating a stronger correlation between predicted and actual values. These observations collectively indicate that GRU-SMA-STL is more robust and reliable, offering lower variability and higher median values for accuracy and predictive power, respectively.

**Figure 7:**
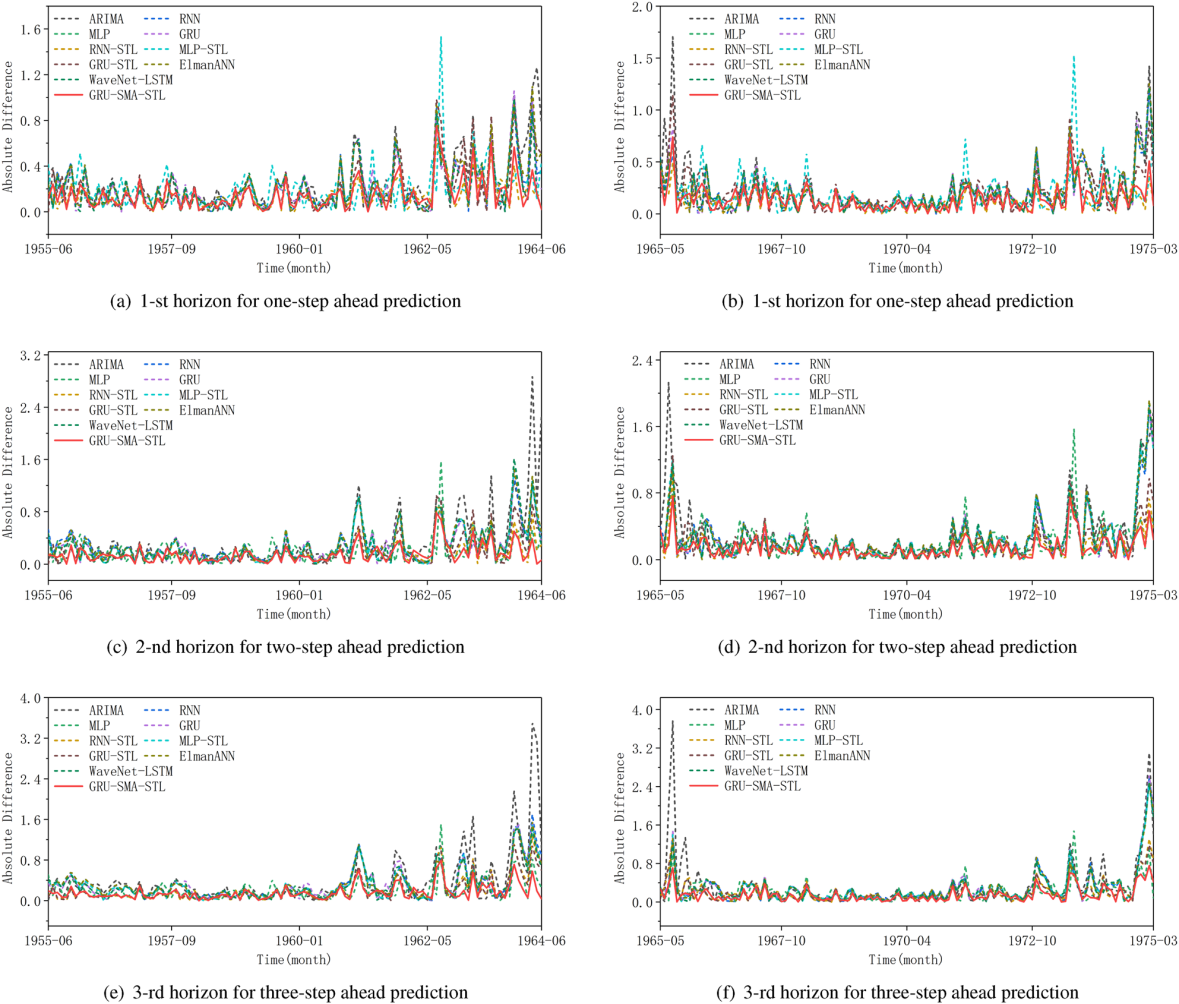
Absolute difference comparison for partial predictions across different models in one-step ahead, two-step ahead, and three-step ahead predictions: (**a**), (**c**), and (**e**) represent the results of the 19-th solar cycle; (**b**), (**d**), and (**f**) represent the results of the 20-th solar cycle.

The comparative performance of different models for sunspot time series prediction may not be adequately captured by simply presenting predicted values. To overcome this limitation, we analyze the absolute difference between actual and forecasted values to provide a more comprehensive comparison of forecasting performance. The results for the first horizon in one-step ahead, the second horizon in two-step ahead, and the third horizon in three-step ahead predictions are presented in Fig. 7 respectively. This approach enhances our understanding of model accuracy and reliability by directly comparing their performance through the magnitude of the absolute difference over the entire forecast period. Our results indicate that the GRU-SMA-STL method consistently exhibits the smallest absolute differences in values across the majority of months, indicating a higher level of agreement between its predicted and actual results. Conversely, the other models display erratic patterns of absolute errors, with sporadic spikes indicating instances of larger prediction errors. This inconsistency suggests that while these models may perform adequately in certain intervals, they are prone to larger errors, possibly due to their inability to adapt to sudden changes or adequately capture the complexity of the time series.

#### Discussion of GRU-SMA-STL guided by pinball loss

We compared the GRU-SMA-STL model, guided by the pinball loss, with the quantile regression model, commonly utilized in probabilistic forecasts. Through a repeated process of 10 iterations, we calculated the average sum of pinball loss for each quantile, ranging from 5 to 95%. A lower loss score indicates a superior probabilistic forecast. The results presented in Table 3 demonstrate that the proposed model improved the average pinball loss by up to 39.44% compared to the benchmark quantile regression model. Based on these findings, we conclude that the GRU-SMA-STL model represents the optimal choice for generating probabilistic sunspot forecasts.

**Table 3:** Overall forecasting performance of two quantile-based models for the sunspot time series.

Forecasting steps	Average pinball loss		Relative improvement (%)
	Pinball loss guided GRU-SMA-STL	Quantile regression	
One-step	4.5586	6.4623	29.46
Two-step	4.5591	7.5278	39.44
Three-step	6.1007	8.0427	24.15

**Figure 8:**
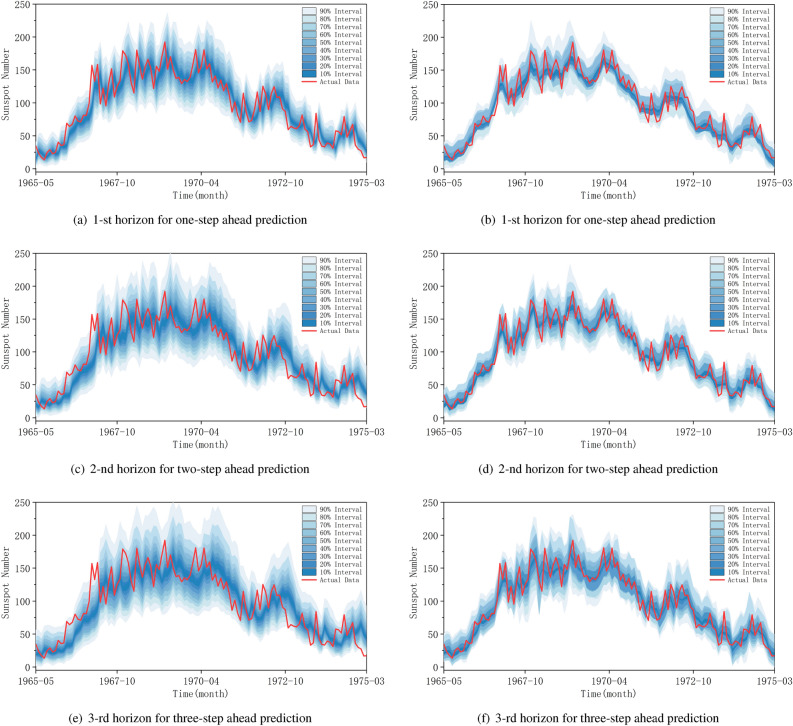
Probabilistic forecasting performance of the 20-th solar cycle: (**a**), (**c**), and (**e**) represent the results of quantile regression; (**b**), (**d**), and (**f**) represent the results of GRU-SMA-STL using pinball loss.

To enhance the interpretability of probabilistic forecasts, we converted the 18 quantiles into nine prediction intervals ($I = 10,..., 90$) with a 10% increment. The prediction interval represents an estimation of the range wherein a forthcoming observation is anticipated to occur. The width of this interval serves as an indicator of the associated uncertainty with the prediction; a narrower interval indicates heightened certainty, whereas a wider interval signifies increased uncertainty. Various factors contribute to the size of the prediction interval, including the confidence level and the model’s accuracy. As the confidence level rises, the interval widens to encompass a broader spectrum of potential outcomes, thereby accommodating a greater degree of variability. The segmented probabilistic forecasts produced by the baseline quantile regression method are depicted in Fig. 8a, c, and e, respectively. The width of the prediction interval is notably influenced by the level of variability in sunspot numbers. Thus, in cases where sunspot number variability exhibits frequent fluctuations, the prediction interval tends to be wider, indicating relatively higher uncertainty in sunspot forecasts. In contrast, Fig. 8b, d, and f present segmented examples of probabilistic sunspot time series forecasts generated by GRU-SMA-STL using the pinball loss over the same periods. Remarkably, the prediction intervals of the proposed model are narrower compared to those of the quantile regression method. The reduced width of the prediction interval signifies less uncertainty in the probabilistic sunspot forecasts obtained through the GRU-SMA-STL method. Consequently, the proposed approach, guided by the pinball loss, outperforms the quantile regression method in providing less uncertain probabilistic sunspot forecasts. Furthermore, the width of the prediction interval increases with the expansion of the prediction horizon. This suggests that a greater number of prediction horizons results in heightened prediction uncertainty. Additionally, the stability of the models diminishes as the prediction horizon extends.

## Conclusion

This paper has introduced a novel combined model, GRU-SMA-STL, which combines slime mould algorithm (SMA) for parameter optimization in the gated recurrent unit (GRU), seasonal-trend decomposition using loess (STL) for time series decomposition, and a unique loss function called pinball loss to guide GRU-SMA-STL training. The methodology involves using SMA to search for optimal parameters for GRU, applying STL to decompose the original sunspot time series into three components (trend, seasonality, and remainder), and utilizing GRU for processing the components and predicting future sunspot values. By using the pinball loss function, the traditional point forecasting of GRU is extended to probabilistic forecasting in the form of quantiles. Evaluation is performed through single-step ahead and multi-step ahead predictions. Results demonstrate that the proposed GRU-SMA-STL model outperforms state-of-the-art methods in the sunspot dataset. The findings underscore the effectiveness of SMA in obtaining suitable parameters for GRU and STL in efficiently addressing the cyclicity of sunspot time series. The use of the pinball loss function with quantile parameters proves effective in handling the uncertainties in sunspot profiles. However, it is acknowledged that the GRU-SMA-STL algorithm has computational cost limitations. Future research directions include exploring modifications to swarm intelligence algorithms to enhance the efficiency of training GRU. We also plan to expand our research to incorporate advanced neural network methods to predict not only sunspot numbers but also the amplitude and timing of solar cycle maxima and minima.

## Data Availability

The data used in this work can be requested from the first author (Z. Cui: cuizhesen@gmail.com).

## Accession codes

A demo of the proposed method in this paper can be obtained by sending a request to the first author.
